# Elevated central venous pressure is associated with increased mortality and acute kidney injury in critically ill patients: a meta-analysis

**DOI:** 10.1186/s13054-020-2770-5

**Published:** 2020-03-05

**Authors:** Chuan-Yu Chen, Yan Zhou, Peng Wang, En-Yao Qi, Wan-Jie Gu

**Affiliations:** 1 Department of Anesthesiology, Luhe People’s Hospital of Nanjing, 9 Jiankang Road, Nanjing, 211500 China; 2 Department of Anesthesiology, Nanjing Drum Tower Hospital, Medical College of Nanjing University, 321 Zhongshan Road, Nanjing, 210008 China

**Keywords:** Central venous pressure, Mortality, Acute kidney injury, Critical illness

## Abstract

**Background:**

The association of central venous pressure (CVP) and mortality and acute kidney injury (AKI) in critically ill adult patients remains unclear. We performed a meta-analysis to determine whether elevated CVP is associated with increased mortality and AKI in critically ill adult patients.

**Methods:**

We searched PubMed and Embase through June 2019 to identify studies that investigated the association between CVP and mortality and/or AKI in critically ill adult patients admitted into the intensive care unit. We calculated the summary odds ratio (OR) and 95% CI using a random-effects model.

**Results:**

Fifteen cohort studies with a broad spectrum of critically ill patients (mainly sepsis) were included. On a dichotomous scale, elevated CVP was associated with an increased risk of mortality (3 studies; 969 participants; OR, 1.65; 95% CI, 1.19–2.29) and AKI (2 studies; 689 participants; OR, 2.09; 95% CI, 1.39–3.14). On a continuous scale, higher CVP was associated with greater risk of mortality (5 studies; 7837 participants; OR, 1.10; 95% CI, 1.03–1.17) and AKI (6 studies; 5446 participants; OR, 1.14; 95% CI, 1.06–1.23). Furthermore, per 1 mmHg increase in CVP increased the odds of AKI by 6% (4 studies; 5150 participants; OR, 1.06; 95% CI, 1.01–1.12). Further analyses restricted to patients with sepsis showed consistent results.

**Conclusions:**

Elevated CVP is associated with an increased risk of mortality and AKI in critically ill adult patients admitted into the intensive care unit.

**Trial registration:**

PROSPERO, CRD42019126381

## Background

Central venous pressure (CVP) is a local hemodynamic parameter determined by the interaction between venous return and cardiac function and used as a surrogate of intravascular volume [1]. Therefore, CVP measurements are often applied for assessing volume status and volume responsiveness to guide fluid resuscitation at the bedside in critically ill patients [2]. However, the validity of CVP in critical care settings has recently been challenged and the use of CVP measurements to direct volume management has been reported unreliable [3]. Based on the rationale provided by the Starling curves and Guyton theory on cardiac function [4], high CVP may impede venous return to the heart and disturb microcirculatory blood flow which may harm organ function, lead to poor prognosis, and even increase mortality. Moreover, elevated CVP will particularly harm kidney hemodynamic and promote acute kidney injury (AKI) by imposing an increased “afterload” on the kidney [5]. However, the association between CVP and mortality and AKI in critically ill patients remains unclear.

So far, previous studies have evaluated the association of CVP and mortality and AKI in critically ill patients but have shown inconsistent results [6–20]. Thus, we performed a meta-analysis to investigate the association of elevated CVP and mortality and AKI in critically ill adult patients, hypothesizing that elevated CVP is associated with increased mortality and acute kidney injury in critically ill adult patients.

## Materials

### Protocol and registration

This meta-analysis was reported in compliance with the PRISMA statement [21]. The protocol was registered on PROSPERO (CRD42019126381). The full details of the protocol are available on request.

### Literature search

Studies were identified by searching electronic databases including PubMed and Embase. No limits were applied for language. We used controlled vocabulary (MeSH in PubMed and Emtree in Embase) and keywords as search terms. The last search was run on June 2019. The full details of the search strategy are available (Additional file 1). In addition, we hand-searched the reference lists of eligible studies.

### Selection criteria

We carried out the initial search, deleted duplicate records, screened the titles and abstracts for relevance, and identified records as included, excluded, or uncertain. In case of uncertainty, the full-text article was acquired to identify eligibility. Studies were eligible for inclusion if they met the following criteria: type of participants, critically ill adult patients admitted into intensive care unit (ICU); type of exposure, CVP; type of outcome, mortality or AKI, defined as individual study (Additional file 2); and types of studies, cohort studies.

### Data extraction

We developed a data extraction sheet in standardized Excel (Microsoft Corporation). One author extracted the following data from included studies and the second author checked the extracted data. The following information was extracted from each study: author, year, country, population, timing of CVP measurement, CVP category, sample size, proportion of mechanical ventilation, event rate, multivariate-adjusted risk estimates, outcomes, study design, and covariates in the fully adjusted model. Any discrepancy was resolved by discussion and consensus.

### Quality assessment

We evaluated the quality of included studies by using the Newcastle–Ottawa scale [22], which is a scale for assessing the quality of observational studies in meta-analyses. This scale awards a maximum of nine stars to each study: four stars for selection of participants and measurement of exposure, two stars for comparability, and three stars for assessment of outcomes and adequacy of follow-up. We assigned scores of 0–3, 4–6, and 7–9 for low, moderate, and high quality of studies, respectively.

### Statistical analysis

The meta-analyses were performed by computing odds ratios (ORs) with 95% CIs for mortality and AKI using a random-effects model, accounting for clinical heterogeneity. Heterogeneity across studies was assessed by using the Q statistic with its *P* value and *I*^2^ statistic [23]. The *I*^2^ statistics used to quantify the proportion of total variation in the effect estimation that is due to between-study variations. An *I*^2^ > 50% indicates significant heterogeneity [24]. A two-sided *P* < 0.05 was considered statistically significant. All analyses were performed using Stata statistical software version 13.0 (StataCorp, USA).

## Results

### Study selection

**Fig. 1:**
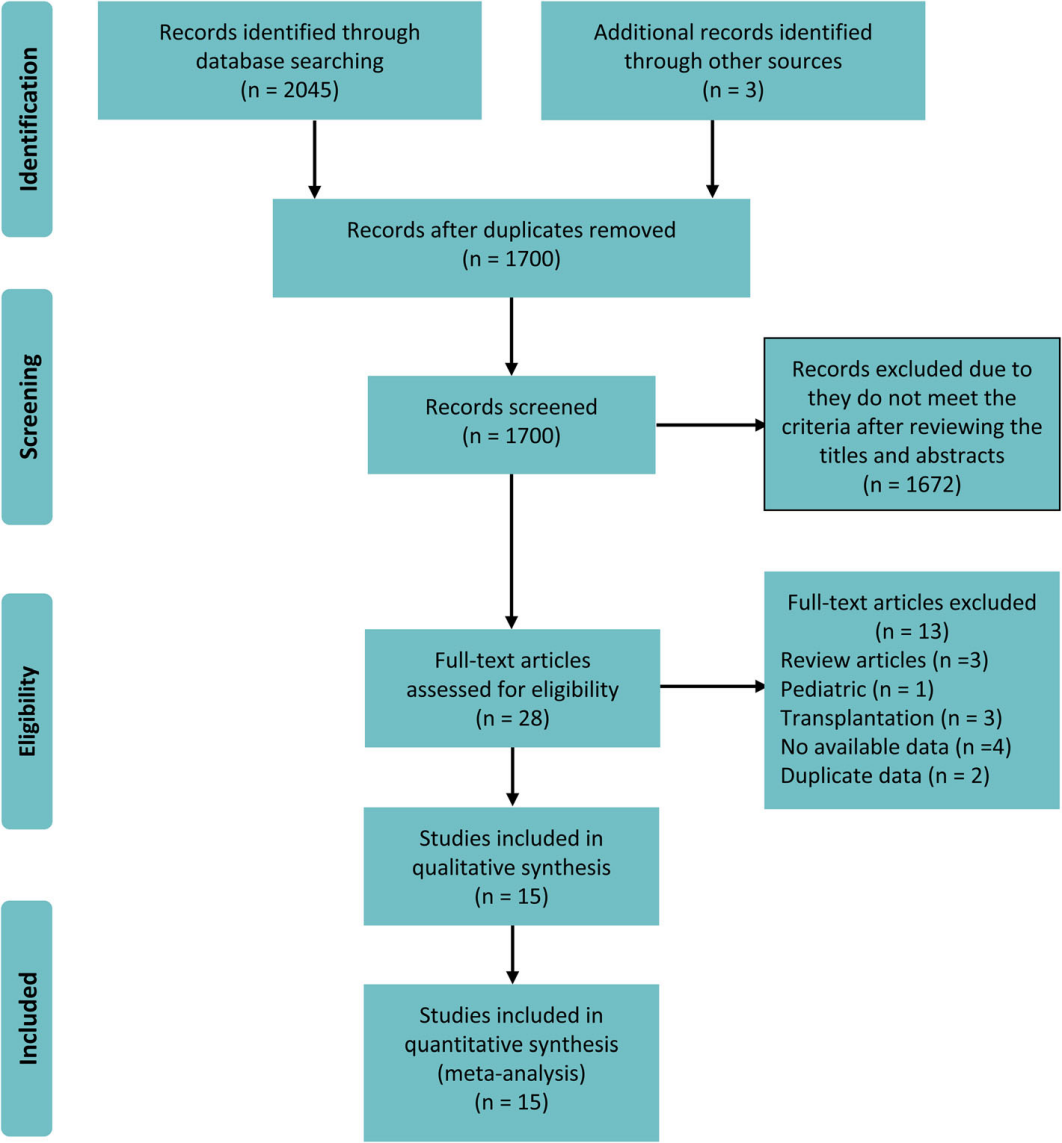
Preferred Reporting Items for Systematic Reviews and Meta-Analyses Flow Diagram

We identified 2048 records in the initial search. After adjusting for duplicates 1700 remained. Of these, 1672 records were discarded because it appeared that these papers clearly did not meet the criteria after reviewing the titles and abstracts. The full text of the remaining 28 articles was examined in more detail. After application of the inclusion criteria, 15 studies were included in the meta-analysis (Fig. 1) [6–20].

### Study characteristics

The characteristics of the included studies are presented in Table 1. These studies were published between 2004 and 2019. The sample size of the individual study ranges from 39 to 4761. The population involved a broad spectrum of critically ill patients (mainly sepsis). CVP category was based on a dichotomous scale and a continuous scale. Adjusted estimates could be determined for almost all studies even though the adjusted factors were slightly different (Additional file 2). Six studies were prospective cohort and the remaining 9 studies were retrospective cohort. Details of the quality assessment of included studies are outlined (Additional file 3). The score for each study was 7 or above, suggesting that all the studies were of high quality.

**Table 1:** Characteristics of included studies

Study	Country	Population	Timing of CVP measurement	CVP category (mm Hg)	Sample size	MV [*n* (%)]	Events [*n* (%)]	OR (95% CI)	Outcomes	Study design
Yegenaga et al. [6]	Belgium	Adults admitted into ICU with SIRS/sepsis	24 h after ICU admission	Continuous	257	209 (81.3%)	29 (11.3%)	1.50 (1.26–1.80)	AKI	PC
Palomba et al. [7]	Brazil	Adults admitted into ICU undergoing cardiac surgery	ICU admission	Dichotomous (> 10 versus ≤ 10)	603	NA	66 (10.9%)	1.92 (1.22–3.03)	AKI	PC
Boyd et al. [8]	Canada	Adults admitted into ICU with septic shock	12 h after ICU admission	Dichotomous (> 12 versus < 8)	778	NA	290 (37.3%)	1.65 (1.10–2.75)	Mortality	RC
				Dichotomous (> 12 versus 8–12)				1.31 (1.06–1.78)		
Chen et al. [9]	China	Adults admitted into ICU with septic shock	24 h after ICU admission	Dichotomous (> 10 versus ≤ 10)	86	NA	29 (33.7%)	2.84 (1.11–7.32)	Mortality	RC
							55 (64.0%)	2.94 (1.18–7.35)	AKI	
Lobo et al. [10]	Brazil	Adults admitted into ICU undergoing high-risk noncardiac surgery	24 h after ICU admission	Continuous	587	NA	121 (20.6%)	1.12 (1.04–1.21)	Mortality	PC
Chung et al. [11]	China	Adults admitted into ICU with severe sepsis or septic shock	ICU admission	Continuous	124	112 (90.3%)	31 (25.0%)	1.15 (1.057–1.251)	Mortality	PC
Legrand et al. [12]	France	Adults admitted into ICU with severe sepsis or septic shock	24 h after ICU admission	Continuous	137	118 (86.1%)	69 (50.4%)	1.23 (1.10–1.38)	AKI	RC
				Continuous (per mmHg increase)				1.22 (1.08–1.39)		
Raimundo et al. [13]	UK	Adults admitted into ICU with stage 1 AKI	12 h after ICU admission	Continuous	210	191 (91.0%)	91 (43.3%)	0.99 (0.94–1.04)	Mortality	RC
Wang et al. [14]	China	Adults admitted into ICU with septic shock	7 days after ICU admission	Dichotomous (> 8 versus ≤ 8)	105	NA	28 (26.7%)	2.70 (1.11–6.58)	Mortality	RC
Wong et al. [15]	Australia	Adults admitted into ICU with septic shock	24 h after ICU admission	Continuous (per mmHg increase)	107	35 (32.7%)	10 (9.3%)	1.28 (1.03–1.60)	AKI	RC
Chen et al. [16]	USA	Adults admitted in ICU with mixed critically ill patients	6 h after ICU admission	Continuous (per mmHg increase)	4761	NA	1095 (23.0%)	1.02 (1.00–1.03)	AKI	RC
Li et al. [17]	China	Adults admitted in ICU with mixed critically ill patients	72 h after ICU admission	Continuous	4708	NA	1179 (25.0%)	1.141 (1.009–1.290)	Mortality	RC
Long et al. [18]	China	Adults admitted in ICU with mixed critically ill patients	24 h after ICU admission	Continuous	2208	2208 (100%)	177 (8.0%)	1.125 (1.069–1.184)	Mortality	RC
Beaubien-Souligny et al. [19]	Canada	Adults admitted into ICU undergoing cardiac surgery	ICU admission	Continuous (per mmHg increase)	145	NA	49 (33.8%)	1.04 (1.01–1.08)	AKI	PC
van den Akker et al. [20]	Netherlands	Adults admitted into ICU with cardiogenic shock	48 h after ICU admission	Continuous	39	28 (71.8%)	24 (61.5%)	1.241 (1.030–1.495)	AKI	PC

*AKI* acute kidney injury, *CI* confidence interval, *CVP* central venous pressure, *ICU* intensive care unit, *MV* mechanical ventilation, *NA* not available, *OR* odds ratio, *PC* prospective cohort, *RC* retrospective cohort

### CVP as a dichotomous scale and mortality and AKI in critically ill patients

**Fig. 2:**
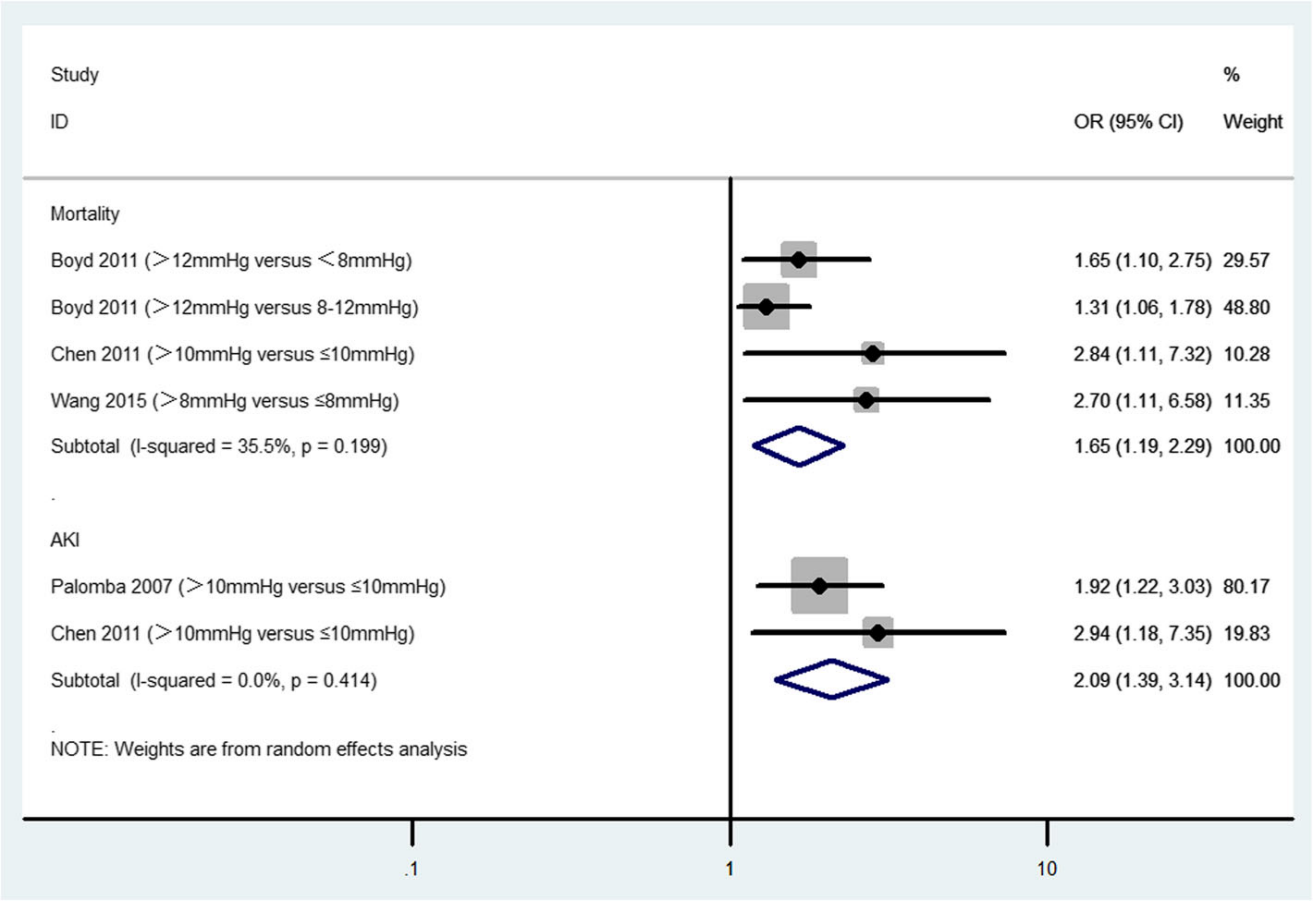
The association of CVP (on a dichotomous scale) and mortality and AKI in critically ill adult patients. CI, confidence interval; OR, odds ratio

On a dichotomous scale, elevated CVP was associated with increased risk of mortality (3 studies; 969 participants; OR, 1.65; 95% CI, 1.19–2.29; *P* = 0.003; *I*^2^ = 35.5%; Fig. 2) and AKI (2 studies; 689 participants; OR: 2.09; 95% CI, 1.39–3.14; *P* < 0.001; *I*^2^ = 0.0%; Fig. 2).

### CVP as a continuous scale and mortality and AKI in critically ill patients

**Fig. 3:**
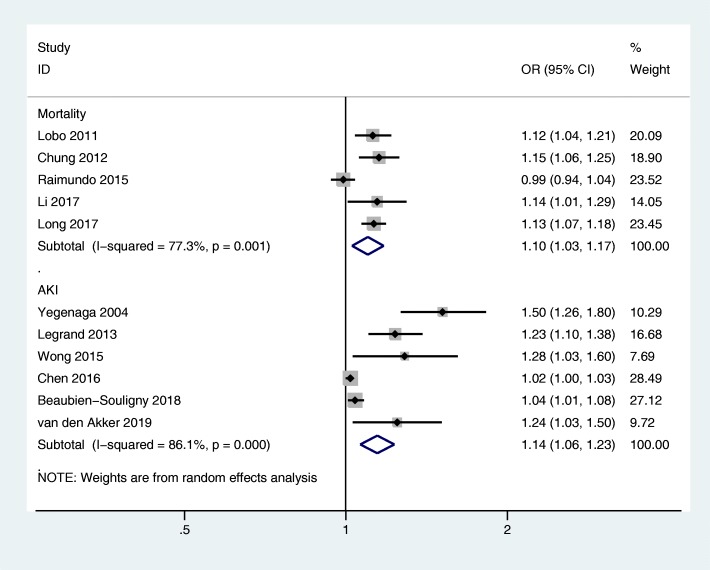
The association of CVP (on a continuous scale) and mortality and AKI in critically ill adult patients. CI, confidence interval; OR, odds ratio

On a continuous scale, higher CVP was associated with greater risk of mortality (5 studies; 7837 participants; OR, 1.10; 95% CI, 1.03–1.17; *P* = 0.006; *I*^2^ = 77.3%; Fig. 3) and AKI (6 studies; 5446 participants; OR, 1.14; 95% CI, 1.06–1.23; *P* < 0.001; *I*^2^ = 86.1%; Fig. 3).

### CVP increase in per 1 mmHg and mortality and AKI in critically ill patients

**Fig. 4:**
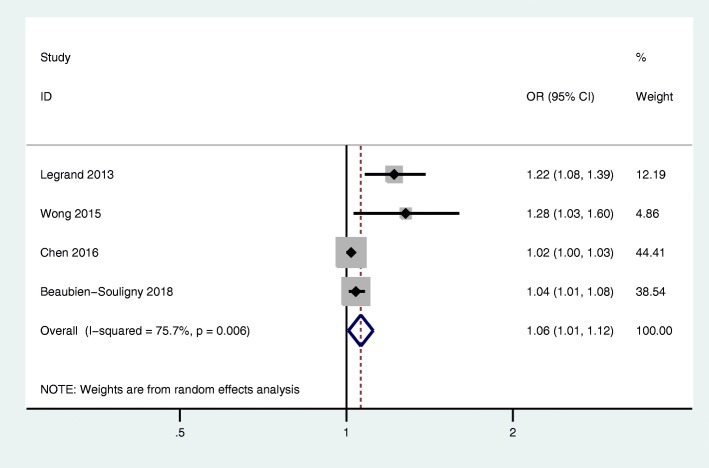
The association of CVP (per 1 mmHg increase) and AKI in critically ill adult patients. CI, confidence interval; OR, odds ratio

No study reported CVP increase in per 1 mmHg and risk of mortality. Four studies with 5150 participants reported CVP increase in per 1 mmHg and risk of AKI. The results suggested that per 1 mmHg increase in CVP increased the odds of AKI by 6% (OR, 1.06; 95% CI, 1.01–1.12; *P* = 0.022; *I*^2^ = 75.7%; Fig. 4).

### Further analyses on CVP and mortality and AKI in patients with sepsis

Three studies with 969 participants reported the association between elevated CVP and risk of mortality on a dichotomous scale. The results suggested that elevated CVP was associated with increased risk of mortality (OR, 1.65; 95% CI, 1.19–2.29; *P* = 0.003; *I*^2^ = 35.5%; Additional file 4). Three studies with 501 participants reported the association between elevated CVP and risk of AKI on a continuous scale. The results suggested that higher CVP was associated with greater risk of AKI (OR, 1.32; 95% CI, 1.16–1.49; *P* < 0.001; *I*^2^ = 41.3%; Additional file 5). Furthermore, per 1 mmHg increase in CVP increased the odds of AKI by 23% (2 studies; 244 participants; OR, 1.23; 95% CI, 1.11–1.38; *P* < 0.001; *I*^2^ = 0.0%; Additional file 6).

## Discussion

### Main findings

To our knowledge, this is the first meta-analysis to investigate the association between elevated CVP and the risk of mortality and AKI in critically ill adult patients. The two principal findings are summarized: elevated CVP is associated with increased risk of mortality and AKI in critically ill adult patients; per 1 mmHg increase in CVP increases the odds of AKI in critically ill adult patients. Further analyses restricted to patients with sepsis showed consistent results.

### Possible mechanisms for findings

CVP is a pressure recorded from the superior vena cava or the right atrium, which represents the pressure index of cardiac preload and is equal to the end-diastolic pressure of the right ventricle in the absence of tricuspid stenosis [25]. It is determined by the interaction between cardiac function and venous return. The CVP is a complex interplay influenced by right ventricular function, right ventricular afterload, right ventricular compliance, venous tone, volume status, abdominal pressure, intrathoracic pressure (mean airway pressure), and many other factors [26]. According to Guyton’s venous reflux theory, cardiac out equals venous return and venous reflux is dependent on mean circulatory filling pressure (MCFP) and CVP gradient (i.e., MCFP-CVP) [4]. Contrary to the misleading assertion that high CVP represents an increase in cardiac output, excessive fluid administration which just leading to an increase of CVP but does not increase cardiac output when the venous return curve intersects this area of the cardiac function curve [26]. This is mainly because fluid loading only increases right atrial pressure/CVP and tissue edema, but does not significantly increase end-diastolic volume and stroke volume on this condition. When CVP increased or MCFP decreased, venous reflux decreased; on the contrary, venous reflux increased if CVP decreased or MCFP increased [27, 28]. Therefore, lower CVP is needed to ensure venous reflux and cardiac output if MCFP in the flat part of the Starling curve.

Traditionally, renal injury is considered to be caused by reduced renal perfusion due to decreased cardiac output or vascular volume [29]. Renal perfusion pressure, defined as mean arterial pressure minus renal venous pressure, is a potential risk factor for AKI progression in critically ill patients [30]. A high CVP is transmitted backwards increasing renal venous pressure, which reduces renal perfusion pressure and increases renal venous congestion [31]. These two factors undependably have profound effects on renal perfusion and renal function and further leading to AKI [32].

### Implications for clinical practice

Our findings have important implications for clinicians to some extent. Almost all patients undergoing major surgery, as well as patients admitted to ICU, will receive CVP monitoring. A normal CVP in a healthy individual is 0–2 mmHg. Legrand et al. found a liner relationship between CVP and the probability of AKI, and increasing the CVP from above 2 mmHg is associated with an increased risk of AKI [12]. In line with this study, our meta-analysis found that per 1 mmHg increase in CVP increases the odds of AKI in critically ill adult patients. It is generally believed that low CVP represents volume depletion and high CVP indicates volume overload; thus, CVP is usually used to make decisions regarding fluid therapy. In fact, to use CVP as a measure of intravascular volume or to define volume responsiveness or the cardiac output response to volume challenges is not only unreliable but also potentially dangerous. In clinical practice, titrating fluid therapy to the CVP is fraught with danger and CVP alone should never be used to make decisions regarding fluid therapy. At the bedside, CVP should be a stopping rule, not a target of fluid resuscitation [33].

### Strengths and limitations

The strength of this meta-analysis lies in compliance with the PRISMA statement and registration on PROSPERO with protocol. Our meta-analysis also has a major limitation that affects the interpretation of the results. The meta-analysis included a broad spectrum of critically ill patients with wide variability. Moreover, there is difference in CVP category, definition of mortality and AKI, and study design. These factors may introduce the heterogeneity and could affect the results. Nevertheless, we used a random-effects model to pool the most fully adjusted estimates, which could reduce the confounding bias in the results.

## Conclusions

Elevated CVP is associated with an increased risk of mortality and AKI in critically ill adult patients admitted into the intensive care unit.

## Supplementary information

**Additional file 1.** Details of Search Strategy.

**Additional file 2.** Definition of Outcomes and Covariates in Fully Adjusted Model.

**Additional file 3.** Quality Assessment of Included Studies by Newcastle–Ottawa Scales.

**Additional file 4.** The association of CVP (on a dichotomous scale) and mortality in patients with sepsis.

**Additional file 5.** The association of CVP (on a continuous scale) and AKI in patients with sepsis.

**Additional file 6.** The association of CVP (per 1 mmHg increase) and AKI in patients with sepsis.

## Data Availability

Not applicable

## Abbreviations

AKI: Acute kidney injury
CI: Confidence interval
CVP: Central venous pressure
OR: Odds ratio
PRISMA: Preferred Reporting Items for Systematic Reviews and Meta-Analyses
