# Supplementary material for: Elevated central venous pressure is associated with increased mortality and acute kidney injury in critically ill patients: a meta-analysis
Source: Crit Care. 2020 Mar 5;24:80. doi: 10.1186/s13054-020-2770-5 (PMC7059303; doi:10.1186/s13054-020-2770-5)
Supplement: Supplementary file 1 — Additional file 1. Details of Search Strategy. [file 13054_2020_2770_MOESM1_ESM.doc]

**Additional file 1 Details of Search Strategy**

**Source: PubMed; Searched on: June 2019; Results: 371**

| **Search** | **Query** | **Items** |
| --- | --- | --- |
| [#1](https://www.ncbi.nlm.nih.gov/pubmed/advanced) | "Central Venous Pressure"[Mesh] | [4434](https://www.ncbi.nlm.nih.gov/pubmed/?cmd=HistorySearch&querykey=10) |
| [#2](https://www.ncbi.nlm.nih.gov/pubmed/advanced) | "central venous pressure"[Title/Abstract] | [6409](https://www.ncbi.nlm.nih.gov/pubmed/?cmd=HistorySearch&querykey=11) |
| [#3](https://www.ncbi.nlm.nih.gov/pubmed/advanced) | CVP[Title/Abstract] | [2994](https://www.ncbi.nlm.nih.gov/pubmed/?cmd=HistorySearch&querykey=12) |
| [**#4**](https://www.ncbi.nlm.nih.gov/pubmed/advanced) | [**#1**](https://www.ncbi.nlm.nih.gov/pubmed/advanced) **OR** [**#2**](https://www.ncbi.nlm.nih.gov/pubmed/advanced) **OR #**[**3**](https://www.ncbi.nlm.nih.gov/pubmed/advanced) | [**10026**](https://www.ncbi.nlm.nih.gov/pubmed/?cmd=HistorySearch&querykey=13) |
| [#5](https://www.ncbi.nlm.nih.gov/pubmed/advanced) | "Renal Insufficiency"[Mesh] | [162593](https://www.ncbi.nlm.nih.gov/pubmed/?cmd=HistorySearch&querykey=16) |
| [#6](https://www.ncbi.nlm.nih.gov/pubmed/advanced) | "Acute Kidney Injury"[Mesh] | [43368](https://www.ncbi.nlm.nih.gov/pubmed/?cmd=HistorySearch&querykey=18) |
| [#7](https://www.ncbi.nlm.nih.gov/pubmed/advanced) | "kidney injury"[Title/Abstract] | [23310](https://www.ncbi.nlm.nih.gov/pubmed/?cmd=HistorySearch&querykey=19) |
| [#8](https://www.ncbi.nlm.nih.gov/pubmed/advanced) | "kidney injuries"[Title/Abstract] | [511](https://www.ncbi.nlm.nih.gov/pubmed/?cmd=HistorySearch&querykey=20) |
| [#9](https://www.ncbi.nlm.nih.gov/pubmed/advanced) | "kidney insufficiency"[Title/Abstract] | [674](https://www.ncbi.nlm.nih.gov/pubmed/?cmd=HistorySearch&querykey=21) |
| [#10](https://www.ncbi.nlm.nih.gov/pubmed/advanced) | "kidney insufficiencies"[Title/Abstract] | [3](https://www.ncbi.nlm.nih.gov/pubmed/?cmd=HistorySearch&querykey=22) |
| [#11](https://www.ncbi.nlm.nih.gov/pubmed/advanced) | "kidney failure"[Title/Abstract] | [7803](https://www.ncbi.nlm.nih.gov/pubmed/?cmd=HistorySearch&querykey=23) |
| [#12](https://www.ncbi.nlm.nih.gov/pubmed/advanced) | "kidney failures"[Title/Abstract] | [28](https://www.ncbi.nlm.nih.gov/pubmed/?cmd=HistorySearch&querykey=24) |
| [#13](https://www.ncbi.nlm.nih.gov/pubmed/advanced) | AKI[Title/Abstract] | [10523](https://www.ncbi.nlm.nih.gov/pubmed/?cmd=HistorySearch&querykey=25) |
| [#14](https://www.ncbi.nlm.nih.gov/pubmed/advanced) | "renal injury"[Title/Abstract] | [10228](https://www.ncbi.nlm.nih.gov/pubmed/?cmd=HistorySearch&querykey=26) |
| [#15](https://www.ncbi.nlm.nih.gov/pubmed/advanced) | "renal injuries"[Title/Abstract] | [964](https://www.ncbi.nlm.nih.gov/pubmed/?cmd=HistorySearch&querykey=27) |
| [#16](https://www.ncbi.nlm.nih.gov/pubmed/advanced) | "renal insufficiency"[Title/Abstract] | [22320](https://www.ncbi.nlm.nih.gov/pubmed/?cmd=HistorySearch&querykey=28) |
| [#17](https://www.ncbi.nlm.nih.gov/pubmed/advanced) | "renal insufficiencies"[Title/Abstract] | [70](https://www.ncbi.nlm.nih.gov/pubmed/?cmd=HistorySearch&querykey=29) |
| [#18](https://www.ncbi.nlm.nih.gov/pubmed/advanced) | "renal failure"[Title/Abstract] | [87106](https://www.ncbi.nlm.nih.gov/pubmed/?cmd=HistorySearch&querykey=30) |
| [#19](https://www.ncbi.nlm.nih.gov/pubmed/advanced) | "renal failures"[Title/Abstract] | [155](https://www.ncbi.nlm.nih.gov/pubmed/?cmd=HistorySearch&querykey=31) |
| [**#20**](https://www.ncbi.nlm.nih.gov/pubmed/advanced) | [**#5**](https://www.ncbi.nlm.nih.gov/pubmed/advanced) **OR** [**#6**](https://www.ncbi.nlm.nih.gov/pubmed/advanced) **OR #**[**7**](https://www.ncbi.nlm.nih.gov/pubmed/advanced) **OR** [**#8**](https://www.ncbi.nlm.nih.gov/pubmed/advanced) **OR** [**#9**](https://www.ncbi.nlm.nih.gov/pubmed/advanced) **OR #**[**10**](https://www.ncbi.nlm.nih.gov/pubmed/advanced) **OR** [**#1**](https://www.ncbi.nlm.nih.gov/pubmed/advanced)**1 OR** [**#12**](https://www.ncbi.nlm.nih.gov/pubmed/advanced) **OR #1**[**3**](https://www.ncbi.nlm.nih.gov/pubmed/advanced) **OR** [**#1**](https://www.ncbi.nlm.nih.gov/pubmed/advanced)**4 OR** [**15**](https://www.ncbi.nlm.nih.gov/pubmed/advanced) **OR #**[**16**](https://www.ncbi.nlm.nih.gov/pubmed/advanced) **OR** [**#1**](https://www.ncbi.nlm.nih.gov/pubmed/advanced)**7 OR #**[**18**](https://www.ncbi.nlm.nih.gov/pubmed/advanced) **OR #**[**19**](https://www.ncbi.nlm.nih.gov/pubmed/advanced) | [**232853**](https://www.ncbi.nlm.nih.gov/pubmed/?cmd=HistorySearch&querykey=32) |
| [#21](https://www.ncbi.nlm.nih.gov/pubmed/advanced) | "Mortality"[Mesh] | [361078](https://www.ncbi.nlm.nih.gov/pubmed/?cmd=HistorySearch&querykey=35) |
| [#22](https://www.ncbi.nlm.nih.gov/pubmed/advanced) | "Death"[Mesh] | [143182](https://www.ncbi.nlm.nih.gov/pubmed/?cmd=HistorySearch&querykey=36) |
| [#23](https://www.ncbi.nlm.nih.gov/pubmed/advanced) | "Survival"[Mesh] | [4609](https://www.ncbi.nlm.nih.gov/pubmed/?cmd=HistorySearch&querykey=37) |
| [#24](https://www.ncbi.nlm.nih.gov/pubmed/advanced) | mortality[Title/Abstract] | [702864](https://www.ncbi.nlm.nih.gov/pubmed/?cmd=HistorySearch&querykey=38) |
| [#25](https://www.ncbi.nlm.nih.gov/pubmed/advanced) | mortalities[Title/Abstract] | [8984](https://www.ncbi.nlm.nih.gov/pubmed/?cmd=HistorySearch&querykey=39) |
| [#26](https://www.ncbi.nlm.nih.gov/pubmed/advanced) | death[Title/Abstract] | [665044](https://www.ncbi.nlm.nih.gov/pubmed/?cmd=HistorySearch&querykey=40) |
| [#27](https://www.ncbi.nlm.nih.gov/pubmed/advanced) | deaths[Title/Abstract] | [163056](https://www.ncbi.nlm.nih.gov/pubmed/?cmd=HistorySearch&querykey=41) |
| [#28](https://www.ncbi.nlm.nih.gov/pubmed/advanced) | survival[Title/Abstract] | [873038](https://www.ncbi.nlm.nih.gov/pubmed/?cmd=HistorySearch&querykey=42) |
| [**#29**](https://www.ncbi.nlm.nih.gov/pubmed/advanced) | [**#21**](https://www.ncbi.nlm.nih.gov/pubmed/advanced) **OR #22 OR #23 OR #24 OR #25 OR #**[**26**](https://www.ncbi.nlm.nih.gov/pubmed/advanced) **OR #27 OR #28** | [**2168787**](https://www.ncbi.nlm.nih.gov/pubmed/?cmd=HistorySearch&querykey=43) |
| [**#30**](https://www.ncbi.nlm.nih.gov/pubmed/advanced) | [**#20**](https://www.ncbi.nlm.nih.gov/pubmed/advanced) **OR #29** | [**2168787**](https://www.ncbi.nlm.nih.gov/pubmed/?cmd=HistorySearch&querykey=43) |
| [#31](https://www.ncbi.nlm.nih.gov/pubmed/advanced) | "Critical Care"[Mesh] | [54579](https://www.ncbi.nlm.nih.gov/pubmed/?cmd=HistorySearch&querykey=47) |
| [#32](https://www.ncbi.nlm.nih.gov/pubmed/advanced) | "Intensive Care Units"[Mesh] | [77794](https://www.ncbi.nlm.nih.gov/pubmed/?cmd=HistorySearch&querykey=48) |
| [#33](https://www.ncbi.nlm.nih.gov/pubmed/advanced) | "intensive care"[Title/Abstract] | [133020](https://www.ncbi.nlm.nih.gov/pubmed/?cmd=HistorySearch&querykey=49) |
| [#34](https://www.ncbi.nlm.nih.gov/pubmed/advanced) | "Critical Illness"[Mesh] | [26065](https://www.ncbi.nlm.nih.gov/pubmed/?cmd=HistorySearch&querykey=51) |
| #35 | "critical care"[Title/Abstract] | [27894](https://www.ncbi.nlm.nih.gov/pubmed/?cmd=HistorySearch&querykey=50) |
| [#36](https://www.ncbi.nlm.nih.gov/pubmed/advanced) | ICU[Title/Abstract] | [50460](https://www.ncbi.nlm.nih.gov/pubmed/?cmd=HistorySearch&querykey=52) |
| [#37](https://www.ncbi.nlm.nih.gov/pubmed/advanced) | "critically ill"[Title/Abstract] | [40783](https://www.ncbi.nlm.nih.gov/pubmed/?cmd=HistorySearch&querykey=53) |
| #38 | "critical illness"[Title/Abstract] | [8138](https://www.ncbi.nlm.nih.gov/pubmed/?cmd=HistorySearch&querykey=54) |
| [**#39**](https://www.ncbi.nlm.nih.gov/pubmed/advanced) | [**#31**](https://www.ncbi.nlm.nih.gov/pubmed/advanced) **OR #32 OR #33 OR #34 OR #35 OR #**[**36**](https://www.ncbi.nlm.nih.gov/pubmed/advanced) **OR #37 OR #38** | [**218120**](https://www.ncbi.nlm.nih.gov/pubmed/?cmd=HistorySearch&querykey=55) |
| [**#40**](https://www.ncbi.nlm.nih.gov/pubmed/advanced) | **#4 AND #30 AND #39** | [**371**](https://www.ncbi.nlm.nih.gov/pubmed/?cmd=HistorySearch&querykey=56) |

**Source: Embase; Searched on: June 2019; Results: 1674**

| **Search** | **Query** | **Items** |
| --- | --- | --- |
| [#1](https://www.ncbi.nlm.nih.gov/pubmed/advanced) | 'central venous pressure'/exp | [12660](https://www.ncbi.nlm.nih.gov/pubmed/?cmd=HistorySearch&querykey=10) |
| [#2](https://www.ncbi.nlm.nih.gov/pubmed/advanced) | 'central venous pressure':ab,ti | [8570](https://www.ncbi.nlm.nih.gov/pubmed/?cmd=HistorySearch&querykey=11) |
| [#3](https://www.ncbi.nlm.nih.gov/pubmed/advanced) | 'cvp':ab,ti | [5458](https://www.ncbi.nlm.nih.gov/pubmed/?cmd=HistorySearch&querykey=12) |
| [**#4**](https://www.ncbi.nlm.nih.gov/pubmed/advanced) | [**#1**](https://www.ncbi.nlm.nih.gov/pubmed/advanced) **OR** [**#2**](https://www.ncbi.nlm.nih.gov/pubmed/advanced) **OR #**[**3**](https://www.ncbi.nlm.nih.gov/pubmed/advanced) | [**17974**](https://www.ncbi.nlm.nih.gov/pubmed/?cmd=HistorySearch&querykey=13) |
| [#5](https://www.ncbi.nlm.nih.gov/pubmed/advanced) | 'kidney failure'/exp | [384560](https://www.ncbi.nlm.nih.gov/pubmed/?cmd=HistorySearch&querykey=16) |
| [#6](https://www.ncbi.nlm.nih.gov/pubmed/advanced) | 'acute kidney failure'/exp | [79886](https://www.ncbi.nlm.nih.gov/pubmed/?cmd=HistorySearch&querykey=18) |
| [#7](https://www.ncbi.nlm.nih.gov/pubmed/advanced) | 'kidney injury':ab,ti | [3622](https://www.ncbi.nlm.nih.gov/pubmed/?cmd=HistorySearch&querykey=19)3 |
| [#8](https://www.ncbi.nlm.nih.gov/pubmed/advanced) | 'kidney injuries':ab,ti | 608 |
| [#9](https://www.ncbi.nlm.nih.gov/pubmed/advanced) | 'kidney insufficiency':ab,ti | [680](https://www.ncbi.nlm.nih.gov/pubmed/?cmd=HistorySearch&querykey=21) |
| [#10](https://www.ncbi.nlm.nih.gov/pubmed/advanced) | 'kidney insufficiencies':ab,ti | [3](https://www.ncbi.nlm.nih.gov/pubmed/?cmd=HistorySearch&querykey=22) |
| [#11](https://www.ncbi.nlm.nih.gov/pubmed/advanced) | 'kidney failure':ab,ti | [9256](https://www.ncbi.nlm.nih.gov/pubmed/?cmd=HistorySearch&querykey=23) |
| [#12](https://www.ncbi.nlm.nih.gov/pubmed/advanced) | 'kidney failures':ab,ti | [41](https://www.ncbi.nlm.nih.gov/pubmed/?cmd=HistorySearch&querykey=24) |
| [#13](https://www.ncbi.nlm.nih.gov/pubmed/advanced) | 'renal injury':ab,ti | [14123](https://www.ncbi.nlm.nih.gov/pubmed/?cmd=HistorySearch&querykey=26) |
| [#14](https://www.ncbi.nlm.nih.gov/pubmed/advanced) | 'renal injuries':ab,ti | 1252 |
| [#15](https://www.ncbi.nlm.nih.gov/pubmed/advanced) | 'renal insufficiency':ab,ti | [30160](https://www.ncbi.nlm.nih.gov/pubmed/?cmd=HistorySearch&querykey=28) |
| [#16](https://www.ncbi.nlm.nih.gov/pubmed/advanced) | 'renal insufficiencies':ab,ti | [109](https://www.ncbi.nlm.nih.gov/pubmed/?cmd=HistorySearch&querykey=29) |
| [#17](https://www.ncbi.nlm.nih.gov/pubmed/advanced) | 'renal failure':ab,ti | [121340](https://www.ncbi.nlm.nih.gov/pubmed/?cmd=HistorySearch&querykey=30) |
| [#18](https://www.ncbi.nlm.nih.gov/pubmed/advanced) | 'renal failures':ab,ti | [276](https://www.ncbi.nlm.nih.gov/pubmed/?cmd=HistorySearch&querykey=31) |
| [**#19**](https://www.ncbi.nlm.nih.gov/pubmed/advanced) | [**#5**](https://www.ncbi.nlm.nih.gov/pubmed/advanced) **OR** [**#6**](https://www.ncbi.nlm.nih.gov/pubmed/advanced) **OR #**[**7**](https://www.ncbi.nlm.nih.gov/pubmed/advanced) **OR** [**#8**](https://www.ncbi.nlm.nih.gov/pubmed/advanced) **OR** [**#9**](https://www.ncbi.nlm.nih.gov/pubmed/advanced) **OR #**[**10**](https://www.ncbi.nlm.nih.gov/pubmed/advanced) **OR** [**#1**](https://www.ncbi.nlm.nih.gov/pubmed/advanced)**1 OR** [**#12**](https://www.ncbi.nlm.nih.gov/pubmed/advanced) **OR #1**[**3**](https://www.ncbi.nlm.nih.gov/pubmed/advanced) **OR** [**#1**](https://www.ncbi.nlm.nih.gov/pubmed/advanced)**4 OR** [**15**](https://www.ncbi.nlm.nih.gov/pubmed/advanced) **OR #**[**16**](https://www.ncbi.nlm.nih.gov/pubmed/advanced) **OR** [**#1**](https://www.ncbi.nlm.nih.gov/pubmed/advanced)**7 OR #**[**18**](https://www.ncbi.nlm.nih.gov/pubmed/advanced) | [**431970**](https://www.ncbi.nlm.nih.gov/pubmed/?cmd=HistorySearch&querykey=32) |
| [#20](https://www.ncbi.nlm.nih.gov/pubmed/advanced) | 'mortality'/exp | [1015424](https://www.ncbi.nlm.nih.gov/pubmed/?cmd=HistorySearch&querykey=35) |
| [#21](https://www.ncbi.nlm.nih.gov/pubmed/advanced) | 'death'/exp | [705560](https://www.ncbi.nlm.nih.gov/pubmed/?cmd=HistorySearch&querykey=36) |
| [#22](https://www.ncbi.nlm.nih.gov/pubmed/advanced) | 'survival'/exp | [1018275](https://www.ncbi.nlm.nih.gov/pubmed/?cmd=HistorySearch&querykey=37) |
| [#23](https://www.ncbi.nlm.nih.gov/pubmed/advanced) | 'mortality':ab,ti | [1009852](https://www.ncbi.nlm.nih.gov/pubmed/?cmd=HistorySearch&querykey=38) |
| [#24](https://www.ncbi.nlm.nih.gov/pubmed/advanced) | 'mortalities':ab,ti | [12483](https://www.ncbi.nlm.nih.gov/pubmed/?cmd=HistorySearch&querykey=39) |
| [#25](https://www.ncbi.nlm.nih.gov/pubmed/advanced) | 'death':ab,ti | [918804](https://www.ncbi.nlm.nih.gov/pubmed/?cmd=HistorySearch&querykey=40) |
| [#26](https://www.ncbi.nlm.nih.gov/pubmed/advanced) | 'deaths':ab,ti | [232930](https://www.ncbi.nlm.nih.gov/pubmed/?cmd=HistorySearch&querykey=41) |
| [#27](https://www.ncbi.nlm.nih.gov/pubmed/advanced) | 'survival':ab,ti | [1267444](https://www.ncbi.nlm.nih.gov/pubmed/?cmd=HistorySearch&querykey=42) |
| [**#28**](https://www.ncbi.nlm.nih.gov/pubmed/advanced) | [**#20**](https://www.ncbi.nlm.nih.gov/pubmed/advanced) **OR #21 OR #22 OR #23 OR #24 OR #**[**25**](https://www.ncbi.nlm.nih.gov/pubmed/advanced) **OR #26 OR #27** | [**3440057**](https://www.ncbi.nlm.nih.gov/pubmed/?cmd=HistorySearch&querykey=43) |
| [**#29**](https://www.ncbi.nlm.nih.gov/pubmed/advanced) | [**#19**](https://www.ncbi.nlm.nih.gov/pubmed/advanced) **OR #28** | [**3742343**](https://www.ncbi.nlm.nih.gov/pubmed/?cmd=HistorySearch&querykey=43) |
| [#31](https://www.ncbi.nlm.nih.gov/pubmed/advanced) | 'intensive care'/exp | [666762](https://www.ncbi.nlm.nih.gov/pubmed/?cmd=HistorySearch&querykey=47) |
| [#32](https://www.ncbi.nlm.nih.gov/pubmed/advanced) | 'intensive care units'/exp | [171094](https://www.ncbi.nlm.nih.gov/pubmed/?cmd=HistorySearch&querykey=48) |
| [#33](https://www.ncbi.nlm.nih.gov/pubmed/advanced) | 'critically ill patient'/exp | 39620 |
| [#33](https://www.ncbi.nlm.nih.gov/pubmed/advanced) | 'intensive care':ab,ti | [189763](https://www.ncbi.nlm.nih.gov/pubmed/?cmd=HistorySearch&querykey=49) |
| [#34](https://www.ncbi.nlm.nih.gov/pubmed/advanced) | 'critical illness':ab,ti | [11097](https://www.ncbi.nlm.nih.gov/pubmed/?cmd=HistorySearch&querykey=51) |
| #35 | 'critical care':ab,ti | [42289](https://www.ncbi.nlm.nih.gov/pubmed/?cmd=HistorySearch&querykey=50) |
| [#36](https://www.ncbi.nlm.nih.gov/pubmed/advanced) | icu:ab,ti | [102360](https://www.ncbi.nlm.nih.gov/pubmed/?cmd=HistorySearch&querykey=52) |
| [#37](https://www.ncbi.nlm.nih.gov/pubmed/advanced) | 'critically ill':ab,ti | [59978](https://www.ncbi.nlm.nih.gov/pubmed/?cmd=HistorySearch&querykey=53) |
| [**#38**](https://www.ncbi.nlm.nih.gov/pubmed/advanced) | [**#31**](https://www.ncbi.nlm.nih.gov/pubmed/advanced) **OR #32 OR #33 OR #34 OR #35 OR #**[**36**](https://www.ncbi.nlm.nih.gov/pubmed/advanced) **OR #37** | [**863302**](https://www.ncbi.nlm.nih.gov/pubmed/?cmd=HistorySearch&querykey=55) |
| [**#39**](https://www.ncbi.nlm.nih.gov/pubmed/advanced) | **#4 AND #29 AND #38** | [**2001**](https://www.ncbi.nlm.nih.gov/pubmed/?cmd=HistorySearch&querykey=56) |
| [**#40**](https://www.ncbi.nlm.nih.gov/pubmed/advanced) | **#4 AND #29 AND #38 AND [humans]/lim AND [embase]/lim** | **1674** |
