# Supplementary material for: Elevated central venous pressure is associated with increased mortality and acute kidney injury in critically ill patients: a meta-analysis
Source: Crit Care. 2020 Mar 5;24:80. doi: 10.1186/s13054-020-2770-5 (PMC7059303; doi:10.1186/s13054-020-2770-5)
Supplement: Supplementary file 2 — Additional file 2. Definition of Outcomes and Covariates in Fully Adjusted Model. [file 13054_2020_2770_MOESM2_ESM.doc]

**Additional file 2 Definition of Outcomes and Covariates in Fully Adjusted Model**

| **Study** | **Definition of Outcomes** | **Covariates in Fully Adjusted Model** |
| --- | --- | --- |
| Yegenaga et al^6^/2004 | AKI: serum creatinine > 2 mg/dl or oliguria (urine output <400 ml/24 h) | Age, primary hepatic failure, vascular surgical intervention, MAP, DBP, urinary output, creatinine, blood urea nitrogen, glomerular filtration rate, pH, bicarbonate, platelet count, albumin, prothrombin time, use of diuretics, and use of vasopressors |
| Palomba et al^7^/2007 | AKI: serum creatinine > 2 mg/dl or an increase >50% from baseline within 7 days after ICU admission | Age, type of surgery, cardiopulmonary bypass time>120 min, low cardiac output, chronic heart failure, preoperative capillary glucose>140 mg/dl, and creatinine>1.2 mg/dl |
| Boyd et al^8^/2011 | Mortality: 28-day | Age, APACHE II score, and use of vasopressors |
| Chen et al^9^/2011 | Mortality: ICU | NA |
|  | AKI: an increase in serum creatinine level >50% from baseline within 7 days after ICU admission or >0.3 mg/dl within 48 hours after ICU admission, or oliguria (urinary output <0.5 ml/kg/h for 6 h) |  |
| Lobo et al^10^/2011 | Mortality: in-hospital | Age, type of surgery, diabetes, peritonitis, heart rate, lactate, and pH |
| Chung et al^11^/2012 | Mortality: 28-day | Diabetes, APACHE II score, SOFA score, antibiotic adequacy, and MAP, and lactate |
| Legrand et al^12^/2013 | AKI: an increase in serum creatinine level >50% from baseline within 7 days after ICU admission or >0.3 mg/dl within 48 hours after ICU admission, or oliguria (urinary output <0.5 ml/kg/h for 6 h) | Age, hypertension, DBP, creatinine, bilirubin, base deficit, fluid balance, and use of vasopressors |
| Raimundo et al^13^/2015 | Mortality: in-hospital | Age, APACHE II score, SOFA score, and use of vasopressors |
| Wang et al^14^/2015 | Mortality: 28-day | NA |
| Wong et al^15^/2015 | AKI: an increase in serum creatinine level >50% from baseline within 7 days after ICU admission or >0.3 mg/dl within 48 hours after ICU admission, or acute dialysis | BMI, diabetes, APACHE II score, MAP, creatinine, fluid balance, and use of vasopressors |
| Chen et al1^6^/2016 | AKI: an increase in serum creatinine level >50% from baseline within 7 days after ICU admission or >0.3 mg/dl within 48 hours after ICU admission, or acute dialysis | Age, gender, race, ICU type, SOFA score, diabetes, congestive heart failure, hypertension, chronic pulmonary disease, peripheral vascular disease, SBP, DBP, heart rate, temperature, creatinine, medication (angiotensin inhibitor converting enzyme-inhibitor, angiotensin receptor blocker, statin, calcium channel blocker, and diuretics), and pulmonary edema |
| Li et al^17^/2017 | Mortality: 28-day | Age, gender, ethnicity, ICU type, SAPS II score, congestive heart failure, cardiac arrhythmias, hypertension, valvular disease, pulmonary circulation disease, renal failure |
| Long et al^18^/2017 | Mortality: 28-day | APACHE II score, SOFA score, heart rate, MAP, perfusion index, P(v-a)co2, lactate, Spo2, mean airway pressure, peak airway pressure, positive end expiratory pressure, driving pressure, and dynamic compliance |
| Beaubien-Souligny et al^19^/2018 | AKI: an increase in serum creatinine level >50% from baseline within 7 days after ICU admission or >0.3 mg/dl within 48 hours after ICU admission | Gender, BMI, chronic obstructive pulmonary disease, type of surgery |
| van den Akker et al^20^/2019 | AKI: an increase in serum creatinine >0.3 mg/dl within 48 hours after ICU admission | Age, BMI, SOFA score, DBP, MAP, creatinine, and use of vasopressors |

Abbreviations: AKI, acute kidney injury; APACHE II, Acute Physiology and Chronic Health Evaluation; BMI, body mass index; DBP, diastolic blood pressure; ICU, intensive care unit; MAP, mean arterial pressure; NA, not available; SBP, systolic blood pressure; SOFA, Sequential Organ Failure Assessment.
