# Supplementary material for: Elevated central venous pressure is associated with increased mortality and acute kidney injury in critically ill patients: a meta-analysis
Source: Crit Care. 2020 Mar 5;24:80. doi: 10.1186/s13054-020-2770-5 (PMC7059303; doi:10.1186/s13054-020-2770-5)
Supplement: Supplementary file 3 — Additional file 3. Quality Assessment of Included Studies by Newcastle–Ottawa Scales. [file 13054_2020_2770_MOESM3_ESM.doc]

**Additional file 3** **Quality Assessment of Included Studies by Newcastle–Ottawa Scales**

| **Study** | **Selection** | | | | **Comparability** | **Outcome** | | | **Total**  **Score** |
| --- | --- | --- | --- | --- | --- | --- | --- | --- | --- |
|  | **Exposed**  **Cohort** | **Nonexposed**  **Cohort** | **Ascertainment**  **of Exposure** | **Outcome**  **of Interest** |  | **Assessment**  **of Outcome** | **Length of**  **Follow-up** | **Adequacy**  **of Follow-up** |  |
| Yegenaga et al^6^/2004 | * | * | * | * | ** | * | * | * | 9 |
| Palomba et al^7^/2007 | * | * | * | * | ** | * | * | * | 9 |
| Boyd et al^8^/2011 | * | * | * | * | ** | * | * | * | 9 |
| Chen et al^9^/2011 | * | * | * | * | - | * | * | * | 7 |
| Lobo et al^10^/2011 | * | * | * | * | ** | * | * | * | 9 |
| Chung et al^11^/2012 | * | * | * | * | * | * | * | * | 8 |
| Legrand et al^12^/2013 | * | * | * | * | ** | * | * | * | 9 |
| Raimundo et al^13^/2015 | * | * | * | * | ** | * | * | * | 9 |
| Wang et al^14^/2015 | * | * | * | * | - | * | * | * | 7 |
| Wong et al^15^/2015 | * | * | * | * | * | * | * | * | 8 |
| Chen et al1^6^/2016 | * | * | * | * | ** | * | * | * | 9 |
| Li et al^17^/2017 | * | * | * | * | ** | * | * | * | 9 |
| Long et al^18^/2017 | * | * | * | * | * | * | * | * | 8 |
| Beaubien-Souligny et al^19^/2018 | * | * | * | * | * | * | * | * | 9 |
| van den Akker et al^20^/2019 | * | * | * | * | ** | * | * | * | 9 |
