# Supplementary figures and images for: Elevated central venous pressure is associated with increased mortality and acute kidney injury in critically ill patients: a meta-analysis
Source: Crit Care. 2020 Mar 5;24:80. doi: 10.1186/s13054-020-2770-5 (PMC7059303; doi:10.1186/s13054-020-2770-5)

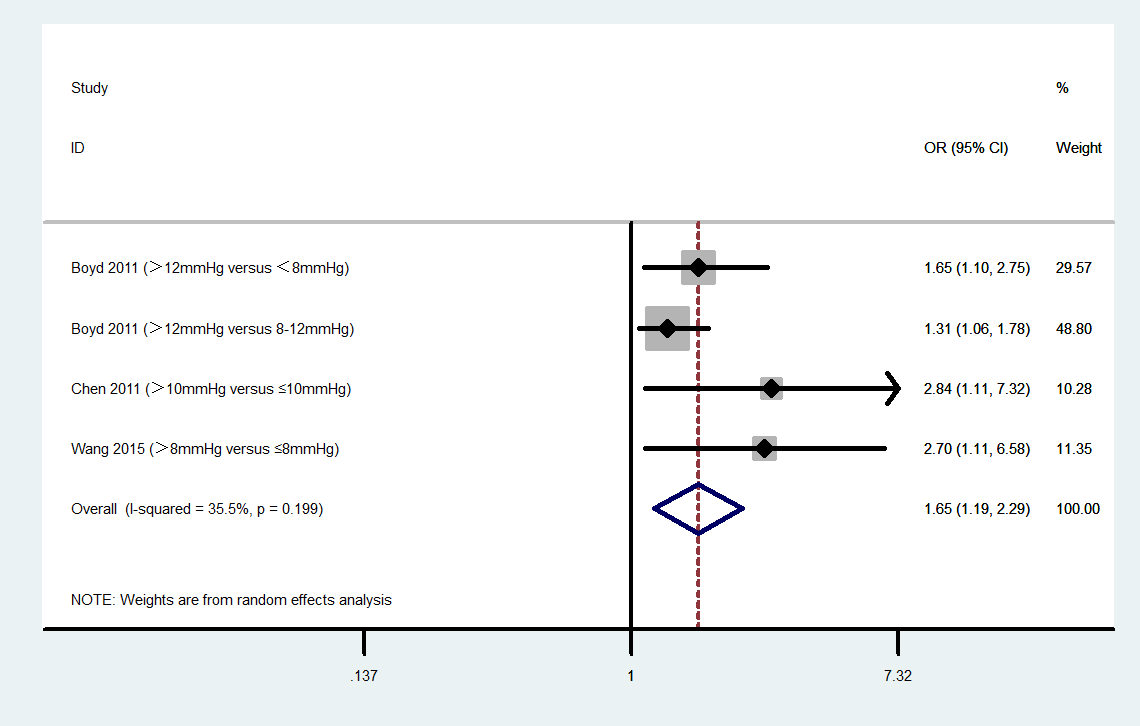

Supplement: Supplementary file 4 — Additional file 4. The association of CVP (on a dichotomous scale) and mortality in patients with sepsis. [file 13054_2020_2770_MOESM4_ESM.png]

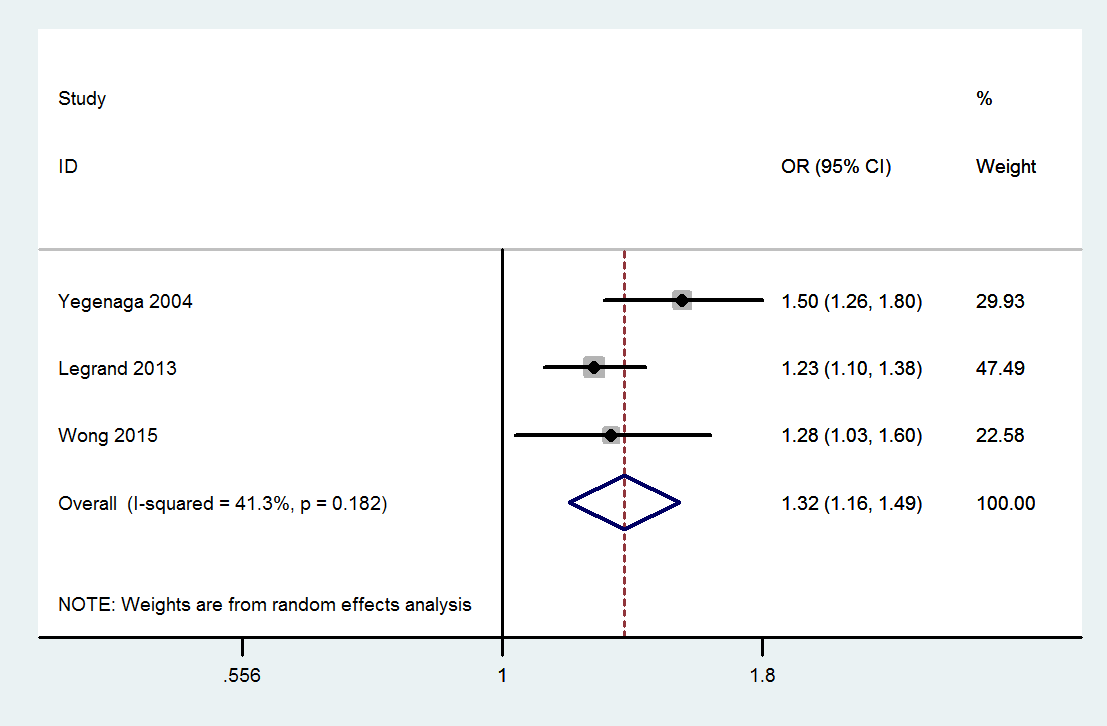

Supplement: Supplementary file 5 — Additional file 5. The association of CVP (on a continuous scale) and AKI in patients with sepsis. [file 13054_2020_2770_MOESM5_ESM.png]

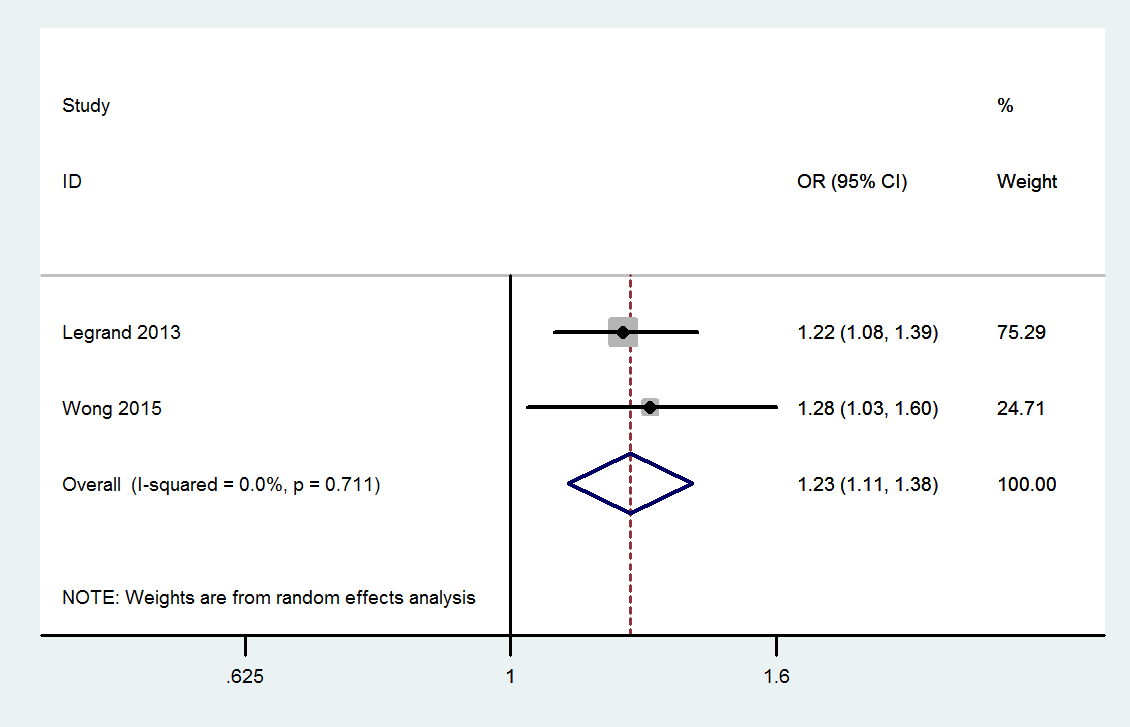

Supplement: Supplementary file 6 — Additional file 6. The association of CVP (per 1 mmHg increase) and AKI in patients with sepsis. [file 13054_2020_2770_MOESM6_ESM.png]
